# Effects of prolonged immunocontraception on the breeding behavior of American bison

**DOI:** 10.1093/jmammal/gyx087

**Published:** 2017-08-10

**Authors:** Calvin L Duncan, Julie L King, Paul Stapp

**Affiliations:** 1 Department of Biological Science, California State University, Fullerton, CA 92831, USA (CLD, PS); 2 Catalina Island Conservancy, Avalon, CA 90704, USA (CLD, JLK)

**Keywords:** *Bison bison*, Catalina Island, immunocontraception, non-lethal population control, PZP, reproductive behavior

## Abstract

In late 2009, the Catalina Island Conservancy began using fertility control to replace periodic removals to manage an introduced population of American bison (*Bison bison*) on the island. Through the application of the immunocontraceptive vaccine porcine zona pellucida (PZP), population growth was slowed within 1 year, and halted over time. In response to lingering questions about the use of PZP to manage large, free-ranging wildlife populations, we sought to determine the reversibility of PZP by ceasing the annual application to a subset of 15 bison cows and monitoring for subsequent calf arrival, and to document changes in the timing and length of the breeding season in response to PZP by monitoring breeding behavior and assessing fecal progesterone (FP) levels for all 60 resident cows over a 13-month period. As of June 2017, no new calves had been observed on the island, suggesting that, following repeated annual treatment with PZP (3 or 4 years), bison do not resume normal reproduction for at least 4 or 5 years, and that fewer treatments would be advisable if a faster return to fertility is desired. Based on observations of bull and cow behavior, and FP levels, cows displayed estrous cycles consistently throughout the study period, indicating that bison may ovulate year-round when conception and its consequences, e.g., lactation and presence of calves, are blocked. Because there is little evidence that an extended breeding season would negatively impact the health of bulls or result in large numbers of out-of-season births on Catalina, PZP appears to be a highly effective tool for managing the population of introduced bison on the island. However, the extended period of contraception and breeding activity of both cows and bulls may make PZP less suitable in high-latitude, predator-rich environments where bison conservation remains a top priority.

Historically, humans have altered wildlife abundance in many ways, including eliminating predators to protect themselves and their livestock, over-harvesting game species, and introducing both non-native and domestic species to new areas where they outcompete or displace native wildlife. The resulting population imbalances require informed management to prevent habitat destruction caused by overgrazing, and the extinction of native species from competition or predation (Asa and Porton 2005). Lethal control, used historically for wildlife species considered pests, is no longer socially acceptable in many parts of the world, and translocation may result in the death of the translocated animals or of other animals in their new range (Conover 2002; Asa and Porton 2005). Translocation efforts can also transport diseases or parasites between populations (Woodford and Rossiter 1993).

Contraception, and especially immunocontraction, is increasingly used for non-lethal control of wildlife populations, especially for species that are non-native or risk overabundance. Using the same principles as disease prevention through vaccination, immunocontraceptive agents are administered in small doses that stimulate the immune system of the target animal to produce antibodies that target and disrupt an essential aspect of reproduction (Kirkpatrick and Turner 1991). Porcine zona pellucida (PZP) vaccine, one of several immunocontraceptive vaccines registered or in development, has been widely used in zoos (Frank et al. 2005) and for free-ranging wildlife (Kirkpatrick and Turner 1991). PZP works by disrupting the zona pellucida, the non-cellular capsule that surrounds all mammalian eggs. This membrane is composed of glycoproteins that act as sperm receptors to facilitate sperm attachment and subsequent fertilization of the egg. When injected into the muscle of the target female, the PZP vaccine (derived from eggs of domestic swine *Sus scrofa domesticus*) stimulates her immune system to produce antibodies against the proteins in the vaccine. These antibodies target the similar protein in the zona pellucida of her eggs, attach to sperm receptors, and thereby block fertilization (Liu et al. 1989; Barber and Fayrer-Hosken 2000). The widespread use of PZP can be attributed to its success in controlling reproduction of wild horses (*Equus caballus*—Kirkpatrick et al. 1990; Turner et al. 1997; Ransom et al. 2011) and white-tailed deer (*Odocoileus virginianus*—Turner et al. 1996; McShea et al. 1997; Miller et al. 1999).

The American bison (*Bison bison*) was nearly driven to extinction by overexploitation (Hornaday 1889). Conservation efforts to recover bison populations continue throughout much of North America, but the growth of localized wild herds in some areas, including locations outside of their native range, has created conflict with human interests and other management objectives (Garrott et al. 1993—Plumb et al. 2009). One such area is Santa Catalina Island (hereafter, Catalina), 1 of the 8 California Channel Islands, United States. In 1924, approximately 14 bison were introduced to the island, and were immediately embraced by the island’s residents as a possible draw for tourists. Genetic variation within the herd was supplemented through the addition of a total of 45 bison between 1934 and 1996, and, at its largest known size in 1987, the herd exceeded 520 individuals (Sweitzer et al. 2005; Duncan et al. 2013). However, in response to the cumulative ecological impacts of introduced mammalian herbivores on the island, beginning in 1969 bison were periodically captured and shipped to the mainland, with the aim of keeping the herd relatively small and minimizing their effects on native plants and animals (Sweitzer et al. 2005).

Since 1972, the Santa Catalina Island Conservancy (hereafter, Conservancy) has been responsible for managing ecological resources on the island, including the bison herd (O’Malley 1994). To better understand their ecological effects, in 2002, the Conservancy commissioned a multiyear population study (Sweitzer et al. 2003) to examine multiple management approaches, all of which included the maintenance of a bison population on Catalina at some level. Sweitzer et al. (2003) suggested that approximately 142 bison could be maintained within their current range of 16,200 ha on the island’s East End, while “providing an acceptable level of protection for the unique natural resources on the island, including endemic plants and animals” (Sweitzer et al. 2003). In an attempt to balance potential ecological impacts with the demand for public viewing opportunities, the Conservancy set a goal of maintaining the herd at approximately 150–200 individuals on the East End (Duncan et al. 2013). However, maintaining this target herd size through occasional removals became unsustainable due to the high costs, concerns about the potential stress on animals during transport, and the expansion of the herd between shipments (Duncan et al. 2013). Additionally, genetic testing suggested a relatively high prevalence of cattle gene introgression in the Catalina herd, which raised concerns about shipping bison to mainland herds with no historical or genetic evidence of hybridization with cattle (Vogel et al. 2007). Given these circumstances, the Conservancy investigated potential methods of fertility control, including immunocontraception, as an alternative to translocation and culling (Duncan et al. 2013). PZP was selected because decades of research on wild horses had demonstrated its safety in pregnant mares, and because it had been shown to increase body condition and longevity in mares treated chronically (Kirkpatrick and Turner 2002, 2003; Turner and Kirkpatrick 2002). The reversibility of PZP, i.e., the ability of an individual to reproduce after cessation of treatment, had also been documented in multiple species, including horses (Liu et al. 1989; Kirkpatrick et al. 1991; Kirkpatrick and Turner 2002), white-tailed deer (Turner et al. 1996), and African elephants (*Loxodonta africana*—Fayrer-Hosken et al. 2000).

Based on its well-documented safety, efficacy and reversibility characteristics, its low cost, and the ability to deliver the vaccine remotely, PZP was first administered to cows in the Catalina herd during 2 roundups in 2009. The first (September 2009) facilitated a final shipment of 149 bison off the island and reduced the island herd to approximately 120 individuals (Table 1). The second (November 2009) supported sample collection, ear tagging, and the application of PZP to approximately 76% of the remaining bison cows (Duncan et al. 2013). The protocol developed by the Science and Conservation Center (Billings, Montana) for species such as bison, with a fairly well-defined breeding season, required a minimum of 2 inoculations during the 1st year of treatment. The 1st inoculation (primer) was administered 1–2 months prior to breeding activity or rut, with the 2nd inoculation 2–6 weeks later, but no later than 1–2 weeks prior to the onset of breeding activity. Infertility could then be maintained by administering a single booster annually, or could be reversed by ceasing booster inoculations (Kirkpatrick and Turner 2002).

**Table 1.** Annual calving and adult mortality rates calculated for a free-ranging bison (*Bison bison*) herd on Catalina Island, California, prior to porcine zona pellucida (PZP) vaccination (2010) and post-PZP vaccination (2011–2016). Data from 2010 to 2012 from Duncan et al. (2013).

Year	Bison population^a^	Calving-age cows (≥ 2 years)	Calves produced	Calving rate (%)	Known mortalities	Mortality rate (%)
2010	120	43	29 (15F:14M)	67.4	5 (1F:4M)	4.2
2011	144	48^b^	5 (5F:0M)	10.4	7 (2F:5M)	4.9
2012	142	61^c^	2 (0F:2M)	3.3	6 (5F:1M)	4.2
2013	138	61^d^	1 (0F:1M)	1.6	3 (1F:2M)	2.2
2014	136	60	0 (0F:0M)	0.0	4 (1F:3M)	2.2
2015	132	59^e^	0 (0F:0M)	0.0	7 (1F:6M)	5.3
2016	125	58	0 (0F:0M)	0.0	5 (1F:4M)	4.0

^a^Island bison population estimate at the beginning of each calendar year.

^b^Four juvenile cows from 2010 reached calving age, 1 unmarked adult cow was discovered.

^c^Two geriatric cows died and 15 female calves from 2010 reached calving age.

^d^Five juvenile cows from 2011 reached calving age and 5 geriatric cows died.

^e^One of the 15 Reversal cows died in December 2014 from a leg injury.

By early 2010, all calving-age cows (≥ 2 years old; *n* = 43) in the population had received the vaccine during the roundups or remotely via darting. Pregnancy testing, completed during the November 2009 roundup, confirmed that 67% (29) of these cows were already pregnant when they received their 1st dose and all successfully produced calves in the spring of 2010 (Duncan et al. 2013). The resulting calving rate of 67.4% (29/43) was identified as the pre-PZP calving potential for the herd. The 2010 calving rate was somewhat higher than that estimated by Sweitzer et al. (2003) in 2001 (49%) and 2002 (35%), presumably due to the reduced herd size (120 versus 303–379 animals) and improved resource availability in 2010. By 2011, the calving rate was reduced to 10.4%, and to 3.3% by 2012 (Table 1). The arrival of calves in 2011 and 2012 was attributed to difficulty in identifying unmarked individuals during early vaccine administration efforts, and a single cow that apparently did not respond to the vaccine during her 1st year of treatment. These problems were resolved through follow-up vaccinations, and the efficacy of PZP in bison on Catalina has been consistently 100% since (Duncan et al. 2013). PZP had been shown to be effective in captive bison (Frank et al. 2005), but our study was the 1st time that PZP had been administered to a large, free-ranging bison herd.

Although the bison contraception program met its initial goal of halting population growth by reducing the calving rate, several key problems remained to be addressed to demonstrate that PZP could be an effective method for long-term, population-wide control of fertility in bison. First, the use of contraceptives to manage high-profile species has raised public concerns about the long-term health of treated populations, and their ability to resume reproduction successfully when the vaccine is no longer administered (Kirkpatrick and Turner 2002). Reversibility has been documented in many but not all species treated with PZP, and the length of time required for the resumption of fertility has varied considerably. On Assateague Island National Seashore, Maryland, Kirkpatrick and Turner (2002) assessed reversibility in wild horse mares after 1–7 consecutive years of treatment with PZP. Of the 53 mares treated with PZP for 1, 2, or 3 consecutive years, 100%, 100%, and 69%, respectively, became fertile within 4 years of the cessation of treatment, and all of the mares that received PZP for 4 or 5 consecutive years returned to fertility within 8 years (Kirkpatrick and Turner 2002). Lyda et al. (2013) showed that female Dall sheep (*Ovis dalli dalli*) and domestic goats (*Capra hircus*) maintained high antibody titers to PZP antigens for 3 years after a single year’s treatment (primer and booster), but that antibody titers in lowland wisent (*Bison bonasus*) and Javan banteng (*Bos javanicus*), species that are more closely related to bison, returned to relatively low levels within only 9 months. Lyda et al. (2013) emphasized the variability among ungulate species and recommended a conservative approach to the application of PZP if a quick return to fertility is desired. In light of this interspecific variability, it was imperative to determine the reversibility of PZP in bison.

A 2nd concern was that PZP would alter the reproductive behavior of bison, specifically, the timing and duration of the breeding season. Adverse changes associated with social behaviors, aggression, and movement and activity patterns have been documented in animals treated with hormone-based fertility control agents (Powell 1999). However, PZP is not hormone-based and the antigen is extremely specific and does not cross-react with other tissues or hormone proteins (Palm et al. 1979; Barber and Fayrer-Hosken 2000). Further, PZP acts upon physiological processes that occur near the end of the reproductive cycle, and therefore does not interfere with other reproductive events (Kirkpatrick et al. 2011). PZP therefore was not expected to elicit behavioral changes as a direct result of vaccine administration. Nonetheless, it is conceivable that the successful act of blocking conception and the absence of calves in the population might alter the behavior of both males and females. These changes may be subtle or inconsequential in captivity, but may be significant for free-ranging populations. Potential concerns include the belief that prolonged or out-of-season breeding behavior as a result of blocked conception and repeated estrous in female ungulates could reduce energy reserves of competing males, especially in populations subject to periods of resource scarcity (McShea et al. 1997; Nuñez et al. 2009; Delsink et al. 2013). Others have speculated that once PZP antibodies decline, offspring could potentially arrive out of season or asynchronously, leaving them more susceptible to adverse weather or predators (Nuñez et al. 2010; Ransom et al. 2013).

Out-of-season breeding activity has been documented in studies of PZP application to free-ranging elk (*Cervus elaphus*—Heilmann et al. 1998) and white-tailed deer (McShea et al. 1997). However, inferences from the elk study are limited because few individuals were treated with PZP, and, in cervids, the breeding season tends to be more regulated by the testicular-antler rhythms of males and is therefore more finite (McShea et al. 1997). Long-term treatment of wild horse mares on Assateague Island did not significantly alter their social organization or behavior (Powell 1999; Kirkpatrick et al. 2011). In other locations, however, PZP-treated mares changed social bands more frequently outside of the breeding season than untreated mares (Nuñez et al. 2009), had different time-activity budgets than untreated ones (Ransom et al. 2010), and cycled and produced foals outside of the normal season (Nuñez et al. 2010; Ransom et al. 2013). Although these studies have been criticized for their small sample sizes and inadequate controls (Kirkpatrick et al. 2011), at minimum they suggest that PZP might alter reproductive behavior of some wildlife species.

In the continental climatic conditions that characterize their native range in the Great Plains of North America, bison generally breed from late June or early July through September, with the majority of breeding activity or copulations occurring from mid-July through mid-August (Meagher 1986). Out-of-season births have been recorded in many bison herds, but the calving season generally extends from early April through early June, with births concentrated from late April to mid-May (Meagher 1986; Pac and Frey 1991; Berger and Cunningham 1994). The timing of the breeding season of bison on Catalina has been poorly documented. Lott and Minta (1983) reported that bison on Catalina initiated breeding much earlier in 1975 (30 May–24 July [55 days]) than in most mainland herds, presumably because of California’s milder Mediterranean climate and seasonality of available resources. More recently, Sweitzer et al. (2003) identified the 90% calving period (the shortest interval in which 90% of the calves were born) in 2002 as occurring over a 3- to 4-week period from mid-April to early May (approximately 24 days), suggesting that, with a gestation period of about 275 days, conception occurred from about 14 July to 7 August 2001. Using a similar approach in 2010, prior to the administration of PZP, we identified the 90% calving period as occurring from 5 March to 17 May 2009 (73 days), suggesting that most conception occurred from early June to mid-August the previous year. These data suggest a somewhat earlier onset and expanded breeding season compared to bison in other locations and from previous research on Catalina; however, a distinct, synchronous calving season has always been apparent. Given the variability between studies, additional data were needed to determine how cessation of conception using PZP would affect breeding behavior and the seasonality of reproduction of bison on Catalina.

Our goals were to investigate the reversibility of PZP in bison after several years of treatment by ceasing administration of the vaccine to a subset of cows and monitoring for new calves, and to determine if the successful blocking of conception by PZP altered the length of the bison breeding season. We hypothesized that bison cows kept infertile for 3 or more consecutive years through PZP treatment would produce calves within 3 years of cessation of booster inoculations. Moreover, at the population level, we predicted that the administration of PZP would not significantly alter the length or seasonality of the breeding season. We interpreted our results in terms of their implications for the continued maintenance of bison on Catalina, and assessed the relevance of our results for efforts to manage bison populations elsewhere.

## Materials and Methods

All animal handling and PZP application procedures were performed under an approved protocol from the Institutional Animal Care and Use Committee at California State University Fullerton, and were consistent with ASM guidelines for research on mammals (Sikes et al. 2016).

### 

#### Study area

**Fig. 1.**
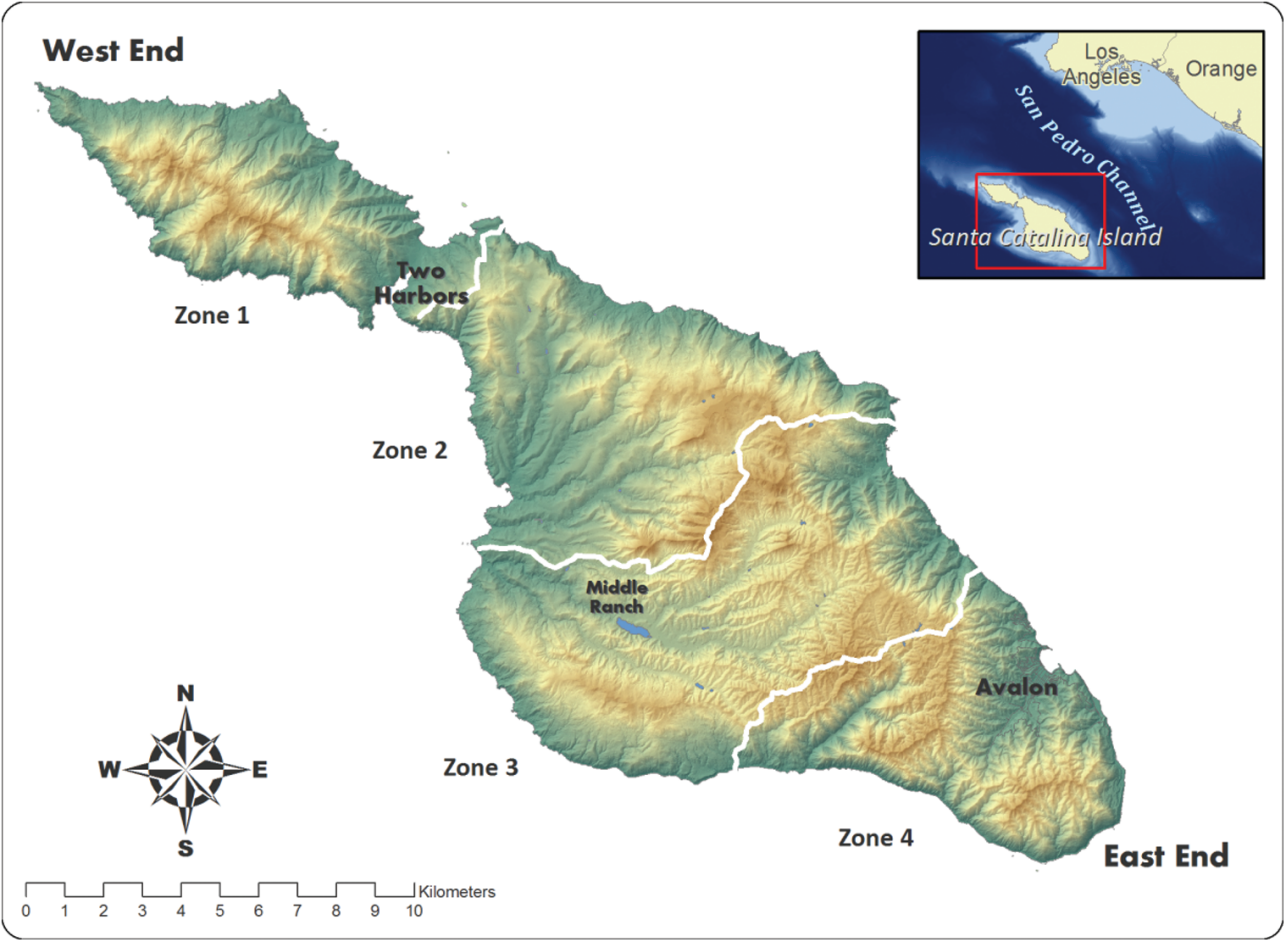
Map of Santa Catalina Island, California, illustrating locations of 3 cross-island fences that partition the island into 4 zones. Zone 1 is free of bison (*Bison bison*), whereas Zones 2, 3, and portions of 4 are occupied by bison. Avalon and Two Harbors are the largest human communities on the island. Inset shows the location of Catalina in relation to mainland southern California.

Santa Catalina Island (194 km^2^), located approximately 40 km west of Los Angeles, California, United States, has a rugged mountainous terrain, with a central ridge running its length (Fig. 1). The elevation ranges from sea level to 640 m, and a narrow isthmus (< 800 m wide) separates the island into 2 geographically distinct sides: the larger East End, comprising 84% of the island, and the smaller West End, comprising the remaining 16% (Schoenherr et al. 1999). The island has a semiarid Mediterranean climate, with average annual precipitation from 2013 to 2015 of 146.6 mm (WRCC 2016), most of which falls between November and April. Common plant communities include coastal sage scrub, coastal bluff scrub, island chaparral, island woodland, riparian woodland, and coastal grassland (Schoenherr et al. 1999). Catalina is home to more than 60 endemic species of plants and animals. It has a resident human population of approximately 4,050, > 90% of which live in the town of Avalon (King et al. 2014), and receives an average of 800,000 visitors annually, making it the most populous of the California Channel Islands.

Although no large mammalian herbivores are native to the island, non-native grazers were introduced over the past 2 centuries. Feral goats were established on the island in the early 1800s, and domestic cattle, horses, and sheep (*Ovis aries*) were present by the 1890s. Feral pigs (*Sus scrofa*) were initially established in the 1930s (Schuyler et al. 1998). Domestic cattle (*Bos taurus*) operations ceased by the 1950s (Schoenherr et al. 1999), and pigs and goats were eradicated between 1990 and 2004 (Schuyler et al. 1998; Sweitzer et al. 2005). Mule deer (*Odocoileus hemionus*) were introduced to the island in 1928 and have been managed since 1998 through a private land management hunting program (Stapp and Guttilla 2006).

#### Breeding behavior and reproductive physiology of bison

Bison are sexually dimorphic, with males (544–907 kg) weighing 60–70% more than females (Meagher 1986). The mating system has been characterized as male dominance polygyny (Lott 1981). Sexual maturity in both female and male bison generally occurs at 2–4 years of age; however, bulls usually do not have the opportunity to breed until age 6 (Meagher 1986). Mixed-sex herds comprised of females and young or subdominant bulls (usually the offspring of cows within the group) may be observed throughout the year. Mature bulls often seek solitude or are found in small bachelor groups during the non-breeding season, and then join the larger cow groups during the rut, when herd sizes are at their peak (Lott 1981). Bulls can identify cows in or near estrus by sniffing or licking their urine or anogenital region and performing *flehmen*, a lip curl and inhalation response that exposes the vomeronasal organ to a scent or pheromone (Mahan et al. 1978; Lott 1981; Mooring et al. 2006).

Once a bull becomes interested in a specific cow, he will tend or guard her, remaining in close proximity, often impeding her movement and warding off other bulls until he is either permitted to copulate with her or is displaced by a rival (Lott 1981; Wolff 1998). Bulls communicate their dominance and desire to mate through posturing, bellowing, pawing, rubbing, scent-urinating, and wallowing (Mooring et al. 2006). A tended cow often attracts rival bulls and head-to-head battles may ensue (Wolff 1998). The breeding season or rut can be particularly taxing for bulls as they may forgo feeding, deplete fat reserves through increased activity, and be injured during battles (Lott 1979; Roden et al. 2011).

Immediately following copulation, which lasts only a few seconds, the cow arches her back, voids a small volume of milky secretions from the vulva (presumably semen), and erects her tail (Lott 1981; Berger and Cunningham 1994; Wolff 1998). Cows elevate their tails for other reasons (urination, defecation, or agitation), but this post-copulation ‘tail-up’ response is distinctive in that the tail is raised at an angle of approximately 135° or higher and is maintained for 1–2 days (Komers et al. 1992; Wolff 1998; Mooring et al. 2006). This behavioral indicator of copulation is very reliable and can be used to infer copulation (Berger and Cunningham 1991; Wolff 1998; Vervaecke and Schwarzenberger 2006). Though uncommon, superficial oval-shaped wounds caused by a mounting bull’s hooves scraping the cow’s back have been observed in recently bred cows and may also be an indicator of breeding (Lott 1981). Cows remain sexually receptive for 9–28 h (Haugen 1974).

Unlike domestic cattle, bison were initially thought to be monestrous (Rutberg 1986) or to only rarely undergo a 2nd estrus in a given year (Haugen 1974). Kirkpatrick et al. (1991a) used urinary steroid metabolites to document definitive estrous cycles of captive bison and reported that 7 of 8 cows became pregnant during their 2nd cycle and subsequently produced calves. The arrival dates for those calves also supported the occurrence of a 2nd seasonal estrus. Vervaecke and Schwarzenberger (2006) used fecal progesterone (FP) metabolites to identify 3 ovulatory periods, and consequently, bison are now considered seasonally polyestrous.

The reproductive physiology of bison is similar to that of domestic cattle. The estrous cycle, as described by Turner (2014), averages 21 days in length (range 17–24). A period of standing heat or estrus, marked by a rise and peak in estrogen secretion, initiates the cycle. Estrus lasts 12–24 h, and ovulation occurs approximately 12 h after the end of estrus. After ovulation, cells that make up the ovulatory follicle differentiate to form the corpus luteum (CL), which, in turn, produces progesterone. Progesterone levels rise substantially and peak after approximately 15–16 days when the CL is fully mature. After approximately 16 days of elevated progesterone, prostaglandin is released by the uterus, resulting in the lysis of luteal tissue and the regression of the CL. Circulating progesterone begins to decline and the luteal phase of the estrous cycle ends. The decline in progesterone triggers increased pulses of gonadotropin-releasing hormone, which, in turn, stimulates the production of follicle-stimulating hormone and luteinizing hormone (LH). Increases in these hormones support the development of another ovulatory follicle, which results in an increase in estrogen. Once estrogen peaks, a surge of LH is released and ovulation occurs again.

Photoperiod has been identified as a cue for reproduction for many wild ungulates, and plant phenology and predation have been implicated as factors contributing to birth synchrony (Rutberg 1984; Green and Rothstein 1993; Zerbe et al. 2012). The breeding season, or rut, of bison has been estimated several ways, such as recording the timing of specific breeding behaviors (tending, copulations, and post-copulation ‘tail-up’ responses of cows), by subtracting gestation length (approximately 265–285 days—Haugen 1974; Rutberg 1986) from parturition, or by monitoring changes in reproductive hormones. Each approach has its own challenges and assumptions. For instance, subtracting gestation length from parturition can provide an estimate of the date of successful conception, but does not yield information on unsuccessful copulation attempts. Brief actions or signs associated with breeding behavior may go undetected when attempting to monitor a large free-ranging population. Assessment of changes in hormones may expand the period of detection, but the collection of adequate blood, urine, or fecal samples systematically from large populations of wild bison can be difficult, dangerous, and expensive. For these reasons, we used a combination of observational and endocrine-based methods to assess changes in the seasonality of breeding behavior in response to PZP application.

#### PZP vaccine preparation and administration

The native PZP antigen was prepared at the Science and Conservation Center from porcine oocytes, using the modified method of Dunbar et al. (1980). Antigen samples were shipped to Catalina on dry ice and stored frozen (−10°C) until time of use, under the authority of the Food and Drug Administration Investigational New Animal Drug Exemption (No. 8840-G003-004). The vaccine consisted of 0.5 ml standard phosphate buffer solution containing 100 µg of PZP emulsified with 0.5 ml Freund’s Modified Adjuvant (FMA; Calbiochem, La Jolla, California) for all initial (primer) inoculations, and 0.5 ml Freund’s Incomplete Adjuvant (FIA; Sigma, St. Louis, Missouri) for all subsequent booster inoculations. The emulsion was prepared within minutes prior to injection using 2 10-ml glass syringes joined with a Luer-Loc connector. After 100 plunger strokes, the emulsion was loaded into 1.0-ml Pneu-Dart type “P” darts with 3.8-cm barbless needles (Pneu-Dart, Williamsport, Pennsylvania) to facilitate remote delivery using an X-Caliber gauged CO_2_ rifle (Pneu-Dart). At the beginning of each darting season, the tail fin of each dart was painted red or orange using Rust-Oleum specialty fluorescent spray paint and numbered to facilitate dart recovery and tracking of vaccinations. Immediately after loading the dart a small dab of petroleum jelly was applied to the tip to prevent PZP leakage. Prepared vaccine was stored in a refrigerator and transported to the field in an ice chest. Treated cows were darted in the caudal thigh or gluteus muscle.

Following the roundup and shipment of bison off-island in late 2009, all calving-age (≥ 2 years old) cows were treated with PZP each year from 2010 to 2012 (Table 2). In 2013, the herd consisted of approximately 138 individuals, 61 of which were calving-age cows. Primer inoculations of PZP + FMA were administered in March 2013 to 5 bison cows that were born in 2011. Booster inoculations of PZP + FIA were administered from February to April 2013, to 51 of 61 cows; from March to April of 2014, to 45 of 60 cows; and from March to April 2015, to 45 of 59 cows (Table 2). These represented all breeding-age cows in the Catalina herd each year. Bison cows were identified through a combination of numbered ear tags (Richey Livestock ID, Brighton, Colorado; applied during previous roundups) and photographs highlighting key features such as horn characteristics, pelage color, and natural markings.

**Table 2.** Annual treatment schedule and delivery method of porcine zona pellucida (PZP) to free-ranging, breeding-aged bison (*Bison bison*) cows on Catalina Island, California, from 2009 to 2012 (Duncan et al. 2013) and 2013 to 2015. The number of treated cows was not necessarily the number of cows in the population that year because cows might have died between January and the darting period. FMA = Freund’s Modified Adjuvant; FIA = Freund’s Incomplete Adjuvant.

Year	Month(s)	Activity	Delivery method	Primer/booster	Adjuvant	Cows
2009	Sep.–Nov.	Roundup	Syringe	Primer	FMA	35
2010	Feb.–May	Field darting	Dart	Primer	FMA	12 of 13^a^
2010	Apr.–Jul.	Field darting	Dart	Booster	FIA	45 of 47^b^
2011	Apr.–Jun.	Field darting	Dart	Booster	FIA	48^c^
2011	Dec.	Roundup	Syringe	Primer/booster	FMA/FIA	13^d^/4
2011	Dec.	Field darting	Dart	Primer	FMA	1^d^
2012	Jan.	Field darting	Dart	Primer	FMA	1^d^
2012	Apr.–May	Field darting	Dart	Booster	FIA	60^e^
2013	Mar.	Field darting	Dart	Primer	FMA	5^f^
2013	Feb.–Apr.	Field darting	Dart	Booster	FIA	51^g^
2014	Mar.–Apr.	Field darting	Dart	Booster	FIA	45^h^
2015	Mar–Apr.	Field darting	Dart	Booster	FIA	45^i^

^a^Additional unmarked adult cow was present but not detected in 2010.

^b^One geriatric cow died early in the year.

^c^One additional adult bison cow was discovered in the population.

^d^Fifteen juvenile cows born in 2010 reached maturity and were added to the program.

^e^Three geriatric cows died between October 2011 and February 2012, and 5 additional geriatric cows died between June and December 2012.

^f^Five juvenile cows born in 2011 reached maturity and were added to the program.

^g^PZP vaccine withheld from 10 adult bison cows (reversibility testing).

^h^One geriatric cow died in late 2013 and PZP vaccine was withheld from 5 additional adult bison cows (reversibility testing).

^i^PZP vaccine withheld from same 14 remaining Reversal cows.

#### Reversibility testing

To assess the reversibility of PZP in bison, the vaccine was withheld from 10 cows (ages 5–11 years) beginning in 2013, and 5 additional cows (ages 8–14 years) in 2014. All but 1 of the Reversal cows were within the range of ages in which the highest fecundity in bison cows has been documented (3–13 years old—Berger and Cunningham 1994). One of the 10 initial Reversal cows died from a leg injury, naturally caused, in late 2014. The vaccine was withheld from the 14 surviving individuals again in 2015, and no cows received PZP in 2016 (Table 2). Reversal cows had received PZP inoculations for 3 (initial 10 cows) or 4 (additional 5 cows) consecutive years; because of the staggered entry (young cows) and exits (deaths) from the program, PZP-treated cows were vaccinated annually a total of 6 times (22 cows), 5 times (2 cows), 4 times (16 cows), and 3 times (5 cows) between 2010 and 2015. All cows in the population were monitored regularly from 2013 through November 2016 to document calf arrival. Using Kirkpatrick and Turner’s (2002) 8-year study of reversibility in wild mares as a guide, we expected approximately 6 (67%) of the initial 9 cows, last treated in 2012, and 1 (20%) of the additional 5 cows, last treated in 2013, to produce calves by the end of 2016.

#### Monitoring of breeding behavior

We monitored behavior and fecal hormone levels of all 60 surviving breeding-aged cows in the Catalina herd as of 1 June 2014. Observations and fecal-sample collections were carried out from a vehicle and on foot over a 13-month period from 1 June 2014 to 30 June 2015. Our aim was to record breeding behavior and signs from all 60 cows every 3–4 days, and to collect a fecal sample from each cow biweekly to determine FP levels. Global positioning system (GPS) collars (G2110E Iridium; Advanced Telemetry Systems, Isanti, Minnesota) were fitted on 5 mature cows on 10–11 May 2014 to assist with locating smaller groups of bison and to assess movement patterns and resource use. Monitoring of the movements of these 5 collared cows allowed us to effectively locate and sample approximately 85–90% of the Catalina herd at all times (Duncan 2017). However, adverse weather conditions, access limitations, safety concerns, and the approachability of the bison all constrained our ability to collect behavioral observations and samples systematically.

For each cow, we recorded the following potential signs of breeding activity (adapted from Vervaecke and Schwarzenberger 2006):

- Tended lightly by bull: A bull, dominant or subdominant but not one of her offspring, remained in close proximity to and followed a cow for longer than 30 min.
- Tended heavily by bull: A dominant bull remained in close proximity to a cow, and often impeded her movements. This activity was accompanied by one or more attempts to mount, cow placing her chin on the bull’s back, and frequent nudging, bellowing, pawing, scent urination, as well as defensive behavior against rival bulls. This level of tending behavior occurred for a prolonged period (> 30 min) and continued until copulation or the end of an observation period.
- Tail-up partial: The cow was observed with a slightly elevated, less than horizontal, tail at the beginning of an observation period and maintained it for 1 h or more.
- Tail-up high: The cow held her tail at a horizontal angle or higher, for an extended period of several hours to several days.
- Copulation: The bull mounted the cow with intromission.
- Fresh mounting sores (FMS): The presence of fresh (new, moist, bloody) wounds on the lower back, point of hip region on either side of a cow.
- Healing mounting sores (HMS): The presence of healing (dry, scabbing) wounds, presumably > 2 days old, on the lower back, in the point of hip region on either side of a cow.

Heavy tending, tail-up high, and copulation behaviors have all been confirmed as reliable indicators of ovulation (Kirkpatrick et al. 1991a; Vervaecke and Schwarzenberger 2006), and were used independently to assess the timing of ovulation in monitored cows. Tail-up partial and light tending were not included as subcategories in previous studies, meaning that these less intensive behaviors were either included within the more general categories of “tending” and “tail-up” or were dismissed altogether. These subcategory behaviors were recorded in the field so that they could be examined separately during analysis, and to help identify individuals that were observed close to the date of ovulation.

#### Collection and analysis of fecal samples

During behavioral observations, cows frequently voided feces. We marked the location of newly voided feces by fixing the location of the feces in the crosshairs of a rifle scope attached to a tripod. We used a range finder to record the distance between the tripod and the target cow. When it was considered safe to approach (within 15 min, on average, and always within 50 min of deposition), a fecal sample was collected to fill about three-quarters of a 30-ml plastic container (Sarstedt Inc., Newton, North Carolina), and placed in a cooler with ice packs. Each collection cup and lid was labeled with the cow’s identification code, date, and time of fecal deposition just prior to sample collection. To ensure a homogeneous sample, fecal material was mixed thoroughly on the ground prior to collection using a single-use wooden tongue depressor. Samples were transferred to a freezer at the end of an observation period and stored frozen (−10°C) until time of shipment. Fecal samples were shipped to the San Diego Zoo Institute for Conservation Research (Escondido, California) within 6 weeks of collection for progesterone radioimmunoassay analysis.

Fecal samples were lyophilized for 72 h in a Flexi-Dry microprocessor manifold lyophiliter (FTS Systems, Inc., Stone Ridge, New York) to reduce variability in water content. Extraneous material was removed from the lyophilized feces by sifting each sample through a copper mesh screen (2 × 2 mm). A 0.1-g sample was weighed and suspended in 5 ml of 90% aqueous ethanol and boiled immediately for 20 min at 80°C. The sample was then centrifuged at 1,000 × *g* for 10 min in a Legend XTR centrifuge (Thermo Fisher Scientific, Waltham, Massachusetts). The supernatant was recovered and the pellet resuspended in 5 ml of 90% aqueous ethanol, vortexed briefly and then centrifuged. The combined supernatants were dried under a stream of filtered air and dissolved in 1 ml of 100% ethanol.

Samples were analyzed using a monoclonal progesterone antibody produced against 4-pregnen-11-ol-3,20-dione hemisuccinate:BSA (Grieger et al. 1990), which cross-reacts 100% with progesterone, 96% with allopregnanolone, and, to a lesser degree, with a number of other progesterone metabolites (Grieger et al. 1990; Wasser et al. 1994). Tritiated progesterone (10,000 cpm/0.1 ml; PerkinElmer, Waltham, Massachusetts) was used to compete against standard progesterone (7–100 pg/0.05 ml; Sigma, St. Louis, Missouri).

One microliter of the fecal extract was assayed in duplicate and incubated overnight at 4°C. After incubation, the competitive reaction was stopped and the bound and unbound hormone were separated by adding 0.25 ml of char-dex solution to the assay, which was then incubated for 30 min at 4°C. The assay was then centrifuged at 1,000 × *g* for 15 min and the supernatant poured into scintillation vials. Scintillation fluid (3.5 ml; MP Biomedicals, Solon, Ohio) was added to each tube and the assay counted on a Beckman LS 6500 liquid scintillation counter (Beckman Coulter, Brea, California) to determine the FP level.

#### Data analysis

We plotted FP levels and breeding behaviors for all 60 cows individually to detect errors and identify possible cycles. For behavioral purposes, a cow was considered to be in estrus if any 1 of 3 characteristic behaviors (heavy tending by bulls, copulation, tail held high) was observed on a given day. To produce a conservative estimate, we considered observations of these behaviors to represent different estrous cycles if they were separated by at least 20 days (Isobe et al. 2005; Turner 2014).

Using the approach of Brown et al. (2001), we estimated a baseline FP value for each cow using an iterative process in which we first calculated the mean and *SD* of all values and then excluded ones that exceeded the mean plus 1.5 *SD*. A new mean was then calculated and these steps were repeated until none of the remaining values exceeded the mean plus 1.5 *SD*. Brown et al. (2001) identified the onset of the luteal phase by the occurrence of FP values that exceeded the baseline by 50% and remained elevated for at least 2 weeks. However, they studied captive animals that were monitored frequently so that increases in FP could be easily identified. To conservatively identify estrous cycles in wild bison cows, we classified FP values as occurring in the luteal phase if they were at least 2 times higher than baseline FP. Given that, in cattle, FP is significantly higher than baseline for approximately 19 days during the estrous cycle (Isobe et al. 2005), we considered elevated FP values to be from different cycles if they were at least 20 days apart.

We were unable to calculate a baseline FP value using the Brown et al. (2001) approach for 8 cows (4 PZP-treated, 4 Reversal). Six of the 8 cows had consistently elevated FP values throughout the study period (71–100% of values > 1,000 ng/g; see “Results”). One cow (Red38) was frequently isolated from the rest of the herd and another (9046) died from a leg injury in December 2014, and so were monitored less frequently. To document the presence of estrous cycles in these 8 cows, we used the mean baseline value (338 ng/g, *SD* = 100) calculated from the other 52 cows. Data from these 8 cows were included in summaries of behavior and FP values, but were excluded from the analysis of temporal patterns in cycles across the herd.

## Results

As of 1 June 2017, no calves had been detected in the herd. This included the 9 surviving Reversal cows that had not been vaccinated since 2012 (60–62 months since last booster; treated for 3 years and off for ~5 years) and the 5 Reversal cows that had not been vaccinated since 2013 (50–51 months since last booster; treated for 4 years and off for ~4 years).

For both behavioral observations and FP sampling, the number of days sampled and the number of total observations and samples collected tended to increase over the course of the 13-month study period (see Supplementary Data SD1). The fewest samples and observations were in summer 2014, whereas, except for a peak in October 2014, the most observations and samples were in spring 2015.

**Fig. 2.**
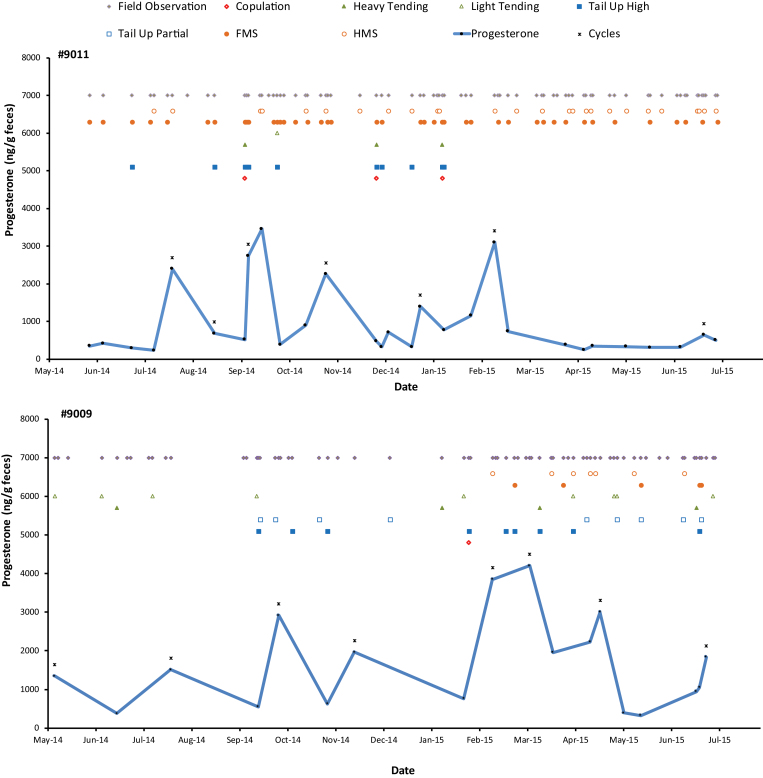
Behavioral and FP profiles of representative PZP-treated and Reversal bison (*Bison bison*) cows on Catalina Island. (top) PZP-treated cow 9011, for which 4 cycles were detected based on behavior, 7 cycles were detected based on FP levels (black stars), with 2 occasions in which behavior and FP overlapped. (bottom) Reversal cow 9009, for which 7 cycles were detected based on behavior, 8 cycles were detected based on FP levels (black stars), with 6 occasions in which behavior and FP overlapped. FP = fecal progesterone; PZP = porcine zona pellucida.

**Fig. 3.**
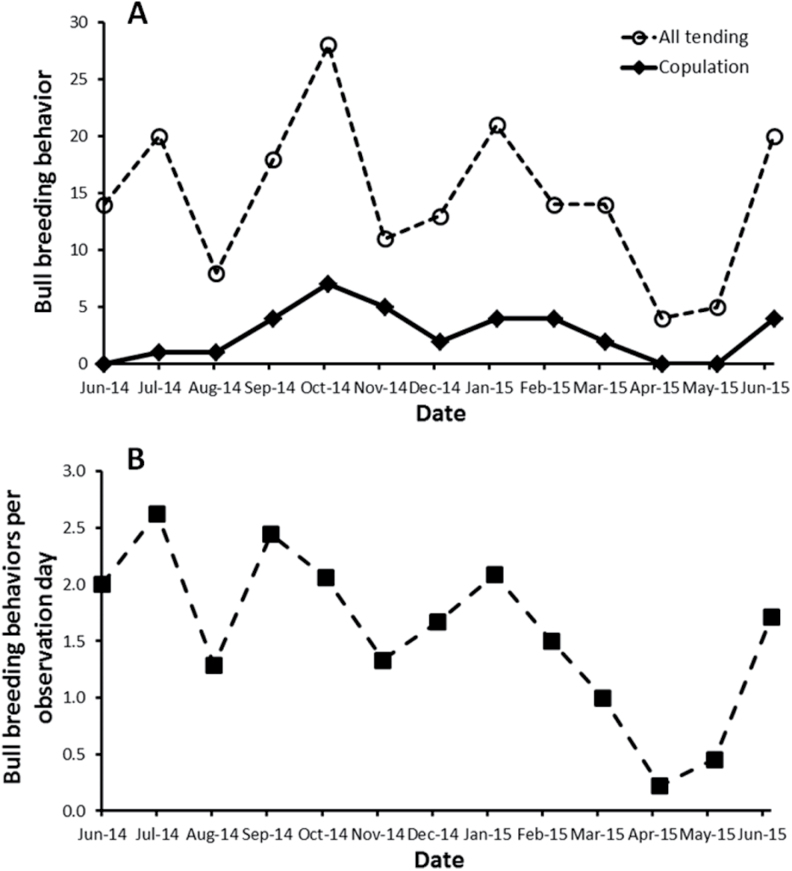
(A) Observations of light and heavy tending and copulations by bison (*Bison bison*) bulls, and (B) all bull breeding behaviors (all tending and copulations) per day of observation, tallied by month, in response to PZP treatment in cows on Catalina Island from 1 June 2014 to 30 June 2015. PZP = porcine zona pellucida.

Breeding behavior and FP levels were plotted together for all 60 cows. Representative profiles of PZP-treated and Reversal cows (see Duncan (2017) for profiles of all cows) are shown in Fig. 2, and a summary of behavioral observations and FP sampling for all 60 cows is given in Appendix I. On average, PZP-treated and Reversal cows were sampled over similar numbers of days (67 versus 61 days, respectively). Reversal cows tended to be heavily tended and copulated more than PZP-treated ones, but the differences were relatively small. For both groups, tail-up high behavior was observed much more frequently than heavy tending or copulations (Appendix I). Fewer than one-half (43%) of all cows were observed copulating, compared to more than 70% that were tended heavily by bulls or observed with their tails held high. Tending behavior by bulls (including light tending) occurred throughout the study period, whereas most copulations were observed between September 2014 and March 2015 (Fig. 3A). Considering all types of breeding behaviors displayed by bulls and adjusting for sampling effort (number of days of behavioral observations per month), bulls apparently were most responsive to females from June 2014 to January 2015, followed by a decline through May 2015, and then increasing again in June 2015 (Fig. 3B).

The presence or absence of mounting sores was determined to not be a good indicator of breeding or ovulation. Although ovulation and breeding behavior were documented in all 60 cows, 20% (12) never developed sores, and sores were rare on many others, despite abundant evidence of breeding activity. Moreover, sores were often persistent and could not be used to indicate the specific timing of copulation. In many instances, sores were re-aggravated or maintained by scratching on trees or bushes, wallowing, or licking, and by mounting activities that were not associated with breeding.

FP values varied greatly within and among individuals (see Supplementary Data SD2). On average, 18.4 samples (range 7–29, *n* = 60) were collected from each cow (Appendix I). Reversal cows tended to have a lower minimum and baseline, and a higher maximum and range of FP values than PZP-treated cows, although these differences were not statistically significant (*t*-tests, *P* > 0.135).

We tentatively identified estrous cycles first based on behavioral observations and FP values separately. Based on behavior alone, on average, Reversal cows displayed more cycles than PZP-treated ones, but the difference was small (2.9 versus 2.5; Appendix I). Using FP levels, Reversal cows had, on average, 1 more cycle than PZP-treated cows (5.9 versus 4.9; Appendix I), but this difference was not significant (*t*_50_ = 2.01, *P* = 0.15). Fewer cycles were identified using behavior than FP values and the 2 measures only occasionally overlapped (Appendix I).

**Fig. 4.**
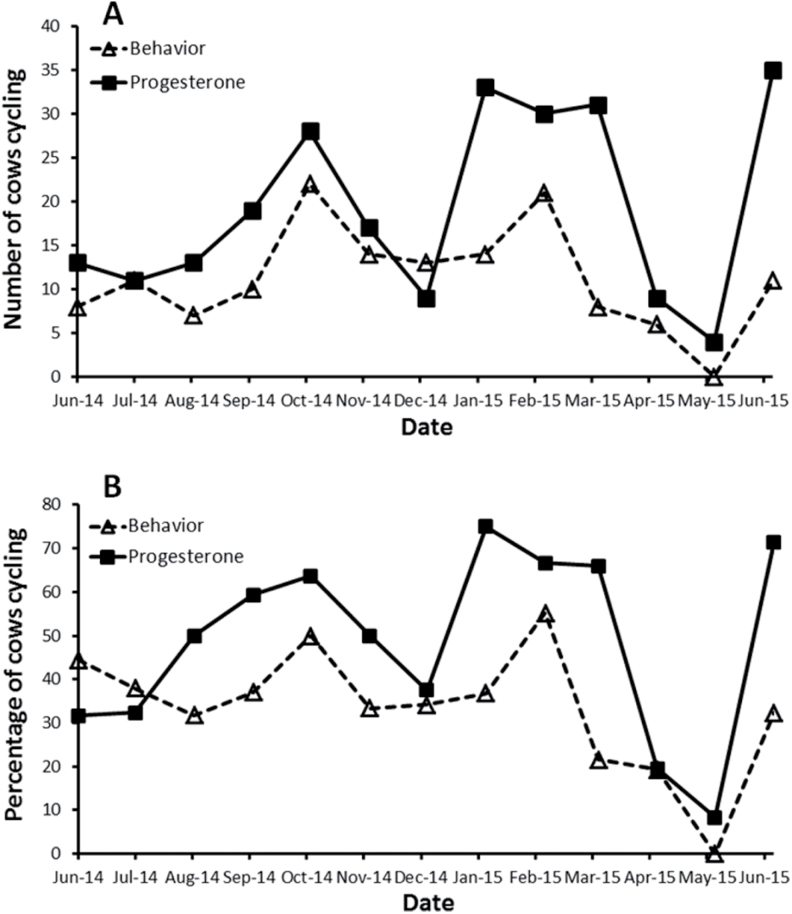
(A) Number and (B) percentage of bison (*Bison bison*) cows displaying estrous cycles, tallied by month, on Catalina Island from 1 June 2014 to 30 June 2015 in response to PZP treatment. Percentage was calculated as a fraction of the number of cows sampled in a given month. Prior to PZP treatment, the typical calving season on Catalina was from March to mid-May, indicating breeding from June to mid-August. PZP = porcine zona pellucida.

To estimate the total number of estrous cycles observed for each cow, we summed the number of behavioral and FP cycles and subtracted the number that overlapped. Considering only the 52 cows with reliable FP data, individual PZP-treated cows displayed 2–12 cycles $(\bar{X}$ = 6.2, *SD* = 2.0, *N* = 41), whereas Reversal cows displayed 4–11 cycles $(\bar{X}$ = 7.3, *SD* = 2.2, *N* = 11; Appendix I), a difference of approximately 1 cycle that was not statistically significant (*t*_50_ = 1.45, *P* = 0.15). Estrous cycles were recorded throughout the 13-month study period (Fig. 4A). Adjusting for the number of cows studied each month (behavior: 18–44; FP: 24–49), a substantial proportion (30–70%) of the resident bison cows were in estrus each month except for April and May 2015 (Fig. 4B).

## Discussion

Administration of PZP to bison cows quickly reduced and, within 3 years, completely halted calf production on Catalina Island, demonstrating for the 1st time the efficacy of PZP as a method of controlling population growth of a large, free-ranging bison herd. Our success can be attributed in part to the fact that the population was closed demographically, that we were able to consistently identify and treat the entire female population, and that the agent’s contraceptive effects were persistent. However, some 4–5 years have passed since PZP was administered to one-fourth (15) of the resident cows and, as of June 2017, no calves have been born on the island. Based on the documented reversibility of PZP in wild horses (Kirkpatrick and Turner 2002), we anticipated that roughly one-half of the Reversal cows would have produced calves by 2016. The shorter (9 months) return to pretreatment PZP antibody titer levels in 2 bovid species closely related to bison (Lyda et al. 2013) also encouraged us that bison might return to fertility relatively quickly. However, Ransom et al. (2013) recently found that the duration of contraception for wild horses increased with the number of years of PZP treatment, such that mares treated with PZP for 3–4 years, like the bison in our study, would not be expected to have their 1st foal until about 5–6 years later. If this pattern applies to bison, calves may not be expected in significant numbers on Catalina for several more years, especially because many cows (24; 40%) were treated for longer (5–6 years) and until as late as 2015. Based largely on our results, the Conservancy elected not to administer PZP boosters to any bison cows in 2016 and will re-assess the administration of PZP when calves are again born on the island.

It is also possible that resumption of fertility in Reversal cows has been delayed because severe drought conditions that persisted on Catalina from 2012 to 2016 reduced the amount and quality of forage. Some of the lowest pregnancy and calving rates recorded in free-ranging bison herds were documented on Catalina Island (35% in 1975 and in 2002) and on Antelope Island, Utah (46% from 1987 to 1997), and in these instances, nutritional deficiencies were suggested as the primary cause (Lott and Galland 1987; Wolfe et al. 1999; Sweitzer et al. 2003). However, the Conservancy provided bison with access to water and modest amounts of supplementary food annually throughout the drought, which should have kept bison in reasonable condition.

Because bison were reported to have at most 2 or 3 estrous cycles in a given year, we anticipated that blocking conception with PZP would, at most, modestly extend the breeding season. Aside from a brief period of low reproductive behavior and progesterone levels in mid-April through May 2015, our results instead suggest that, like domestic cattle (Garverick and Smith 1993), bison are capable of cycling nearly year-round when conception and its consequences, e.g., lactation and presence of calves, are blocked by PZP. This was evident in the behavior of the bulls as well, which continued to copulate with and tend females intensively throughout the year.

Two concerns that have been raised about an extension of the breeding season in PZP-treated ungulates are the potential impacts on the condition of males as a result of an extended rut (Nuñez et al. 2009,^2010^; Delsink et al. 2013), and risks associated with out-of-season calving (McShea et al. 1997; Nuñez et al. 2010; Ransom et al. 2013). A closer examination of the behavior of bison, specifically the frequency of shifts in dominance among bulls, and the survival of offspring born out-of-season in species subject to harsh winters, may help address these concerns, especially in comparison to the consequences of other management approaches such as removals or culling that might be applied to the Catalina herd.

Bison bulls that are 7–13 years old and weigh > 750 kg are the dominant age group, and are responsible for approximately 95% of copulations (Wolff 1998). Both tending and challenging bulls feed less during peak rut than during early rut, lose considerable amounts of weight during rut, and may sustain debilitating or life-threatening injuries during battles (Wolff 1998; Roden et al. 2011). In most herds, dominance rankings among bulls change from year to year as younger bulls mature and grow in size, and begin to challenge older or weaker bulls. Dominance may only be maintained for 2–3 weeks during the rut before an individual is displaced (Wolff 1998). Once a dominant bull is displaced, time off from tending or fighting often allows him to regain enough weight and strength to again challenge for breeding rights (Wolff 1998). Prolonged or continuous ovulation in females therefore may speed up this cycle or the rotation of bulls through periods of dominance, but it may not necessarily affect health or survival of bulls significantly.

Although we did occasionally observe head-to-head battles, in the absence of a long non-breeding period, we speculate that dominance among Catalina bulls was likely maintained through non-contact behaviors such as posturing, bellowing, head swaying, and scent urination, until a challenger reached a size and condition to confidently compete with a tending bull. Abscesses, presumably associated with battles, were occasionally observed, but dominant bulls with more serious injuries were not seen. Due to the high profile of the bison herd on Catalina, and the frequency in which they are observed by Conservancy staff, island tours, and residents, conspicuous injuries, such as a limp or open wound, are unlikely to go undetected. Based on these reports and our own informal observations, we found no evidence that the frequency of injuries or mortalities has increased as a result of the PZP program. Adult bison on Catalina have no predators, and remain brucellosis and tuberculosis free, but they still are subject to marked seasonal variations in the availability of forage and water, which were exacerbated by the drought. Given our close monitoring of the herd since the inception of the PZP program in 2009, any negative effects of an extended rut on Catalina should have been apparent by now.

At the individual level, photoperiod, temperature, vegetation green-up, and female body condition influence the timing of parturition in many ungulate species, whereas resource availability is believed to be the primary driver of birth season length in populations in highly seasonal habitats (Rutberg 1987; Green and Rothstein 1993; English et al. 2012; Zerbe et al. 2012). Because of Catalina’s latitude and coastal position, these factors are mitigated to some extent compared to the Great Plains, but strong seasonal variability still occurs, in terms of pronounced wet and dry seasons. During non-drought periods vegetation green-up on Catalina generally occurs in late winter (January–February), with nutritious vegetation persisting until summer (July–August). As a result, the calving season on the island begins sooner than in most mainland herds (March versus April), but lasts about the same amount of time (Meagher 1986; Pac and Frey 1991). Out-of-season births occur in many herds (Meagher 1986; Berger and Cunningham 1994), and birth synchrony and birth season length have been found to vary due to forage availability (Green and Rothstein 1993) and population size (Meagher 1973). On Catalina, calves have been observed occasionally in September and December (Sweitzer et al. 2003) and in 2010, 2 calves were recorded in mid-February. However, the milder winter and lack of predators poses less of a mortality risk to calves born out of season on Catalina than in much of their native range. In some species, PZP application led to more out-of-season births and changes in birth synchrony (McShea et al. 1997; Nuñez et al. 2010; Ransom et al. 2013), whereas, in others, no changes were seen (Kirkpatrick and Turner 2003; Gray et al. 2010). Because we observed breeding-related behaviors and estrous cycles throughout the year, it is conceivable that declining PZP antibody titers would eventually result in more out-of-season calves on Catalina. However, until bison cows on the island again produce calves, we have no evidence that this occurs.

We underscore that bison are not native to Catalina and that some aspects of their biology on the island are quite different than in their natural range. Because of the special cultural significance of bison to island residents, and their possible role in reducing fuel loads and minimizing damage from wild fires (Sweitzer et al. 2005), it is unlikely that bison will be removed from the island in the foreseeable future. However, the bison contraception program represented an opportunity to reduce the ecological damage caused by a large, historically overabundant herbivore on the island, and our research has shown that PZP is much more cost-effective than shipments of bison off-island (Duncan 2017) and less controversial than culling. In similar circumstances elsewhere, the prolonged period of contraception apparently afforded by PZP may represent an advantage over other methods of population control. Nonetheless, we caution that the differences in climate and predators between coastal southern California and most of the native range of bison may have important implications for the application of PZP to herds elsewhere. For example, immunocontraception has been proposed as a method for reducing transmission of brucellosis in the Greater Yellowstone Area because it could prevent bison cows infected with *Brucella abortus* from becoming pregnant and thus spreading the pathogen to other bison, elk, and livestock by the shedding of infected birthing tissues and fluids and aborted fetuses (Rhyan et al. 2013). APHIS (2012) rejected PZP as a potential contraceptive agent for Yellowstone because of concerns that it would prolong the breeding season, resulting in increased activity by dominant bulls and PZP-treated cows during fall and early winter that increased mortality (Miller et al. 2004). Although the high costs associated with out-of-season breeding activity remain to be elucidated fully, our work confirms that PZP treatment does result in year-round breeding activity of both cows and bulls, making this a legitimate concern in high-latitude, continental climates where large predators are still present.

## Supplementary Data

Supplementary data are available at *Journal of Mammalogy* online.

**Supplementary Data SD1.**—Sampling effort (number of days, numbers of observations, and FP samples collected) on Catalina Island from 1 June 2014 to 30 June 2015.

**Supplementary Data SD2.**—Range of fecal progesterone values for 60 bison (*Bison bison*) cows on Catalina Island between 1 June 2014 and 30 June 2015.

## Supplementary Material

Supplementary Data SD1

Supplementary Data SD2

## Appendix I

Summary of behavioral and fecal progesterone (FP; ng/g) measurements of 60 bison (*Bison bison*) cows on Santa Catalina Island, between 1 June 2014 and 30 June 2015. Duration is the number of days between the 1st and last behavioral observations. Overlap indicates the number of estrous cycles which were identified by both behavior (Behavior) and FP using the Brown et al. (2001) approach. The total number of cycles detected was estimated by summing the number of behavioral and FP cycles and subtracting the instances of overlap. No baseline FP value could be calculated for the last 8 cows (below the *Baseline*), so the number of FP and overlapping cycles were estimated using the mean baseline FP (338 ng/g) for the other 52 cows.

**Table:**

						BEHAVIOR			FP (ng/g)					CYCLES^a^			
Bison ID#	Status	Age in 2014	No. years of PZP treatment	No. days observed	Duration (days)	Heavy tending	Copulation	Tail high	No. samples	Minimum	Baseline^a^	Maximum	Range	Behavior	FP	Overlap	(Behavior + FP) − Overlap
7913	PZP	3	3	67	391	2	0	2	27	246	356	3,138	2,891	3	6	2	7
9003	PZP	6	5	65	396	0	0	1	25	183	304	977	795	1	3	0	4
9004	PZP	10	6	66	397	0	0	0	22	214	299	2,022	1,808	0	5	0	5
9005	PZP	3	3	69	392	1	0	1	15	357	448	3,186	2,829	2	4	0	6
9006	PZP	3	3	69	392	1	1	1	19	225	373	2,557	2,331	2	2	1	3
9007	PZP	5	6	74	391	3	1	2	21	294	355	3,296	3,002	3	8	2	9
9008	PZP	7	6	56	379	5	1	13	14	367	403	3,841	3,474	7	4	3	8
9011^b^	PZP	4	4	77	372	3	3	11	29	227	318	3,460	3,233	4	7	2	9
9012	PZP	3	3	74	392	0	0	3	17	222	370	2,711	2,489	3	3	0	6
9013	PZP	4	4	53	389	0	0	0	13	154	308	5,640	5,487	0	2	0	2
9014	PZP	10	6	92	340	0	0	1	20	173	308	3,348	3,175	1	3	0	4
9016	PZP	4	4	82	388	2	1	3	24	288	417	1,793	1,505	2	5	0	7
9017^b^	PZP	4	4	82	374	3	0	3	22	168	238	2,919	2,751	3	3	1	5
9018	PZP	4	4	74	387	5	0	4	12	203	466	2,114	1,912	5	2	1	6
9019^b^	PZP	4	4	72	392	3	0	0	17	208	473	6,271	6,062	3	5	2	6
9021	PZP	8	6	38	395	1	0	2	12	201	305	1,313	1,111	2	2	0	4
9023	PZP	4	4	81	394	1	0	0	19	64	272	2,395	2,331	1	5	1	5
9025	PZP	4	4	57	391	6	0	0	20	76	264	2,593	2,517	3	5	3	5
9027^b^	PZP	13	6	70	390	1	0	3	18	64	236	2,548	2,484	2	6	2	6
9029^b^	PZP	4	4	73	389	2	1	1	19	67	164	5,689	5,622	2	7	1	8
9030^b^	PZP	4	4	59	349	1	1	4	18	92	349	4,469	4,377	4	6	3	7
9032	PZP	4	6	85	392	3	1	1	23	210	470	6,002	5,792	3	7	1	9
9033	PZP	4	4	55	392	5	0	2	19	63	372	1,302	1,239	4	5	1	8
9036	PZP	4	4	79	392	0	0	0	22	128	316	3,928	3,800	0	7	0	7
9038	PZP	12	6	48	384	0	0	1	18	77	269	3,275	3,198	1	5	0	6
9040	PZP	12	6	69	394	1	1	6	19	58	283	5,419	5,361	4	7	4	7
9041^b^	PZP	5	6	60	387	0	0	1	12	44	285	873	829	1	1	0	2
9042^b^	PZP	5	6	64	389	1	1	1	7	156	497	4,321	4,165	1	3	0	4
9044	PZP	12	6	74	405	0	0	0	24	136	237	2,186	2,050	0	6	0	6
9045	PZP	5	6	62	392	3	0	2	20	57	336	5,042	4,985	4	6	2	8
9047	PZP	10	6	67	405	0	0	7	25	345	592	5,305	4,960	4	7	2	9
9048	PZP	6	6	66	375	6	1	5	10	275	602	3,065	2,790	5	2	2	5
9053^b^	PZP	19	6	75	405	1	0	1	25	223	454	2,787	2,564	1	6	1	6
9054	PZP	9	6	74	390	0	0	1	22	50	205	1,294	1,244	1	6	0	7
9056	PZP	4	4	62	397	2	2	3	16	42	223	2,581	2,539	2	5	2	5
9057	PZP	4	4	65	394	4	1	10	22	153	332	3,371	3,218	6	7	5	8
9058^b^	PZP	4	4	61	382	2	1	3	18	128	362	5,454	5,326	3	5	2	6
9059^b^	PZP	4	4	78	387	1	0	4	23	92	343	4,244	4,152	2	6	1	7
9060	PZP	7	5	77	392	1	1	8	17	60	414	6,994	6,934	3	4	0	7
9061	PZP	9	6	97	397	6	3	13	27	63	203	3,532	3,469	8	9	5	12
UM13	PZP	3	3	80	391	0	0	1	22	159	481	5,197	5,038	1	4	0	5
9001	Reversal	8	3	76	390	1	1	4	17	140	214	5,489	5,348	4	5	3	6
9009	Reversal	8	3	73	405	4	1	9	18	323	507	4,206	3,881	7	8	6	9
9015^b^	Reversal^c^	10	4	53	292	0	0	2	14	212	267	3,014	2,802	2	7	2	7
9020^b^	Reversal	9	3	78	387	2	0	3	28	260	376	4,320	4,060	2	11	2	11
9022^b^	Reversal^c^	10	4	66	394	1	0	3	16	156	217	1,648	1,492	3	4	3	4
9026	Reversal	12	3	60	389	3	1	4	20	119	304	3,758	3,639	4	6	3	7
9037^b^	Reversal	8	3	71	389	5	1	4	17	115	283	3,146	3,031	4	7	2	9
9039^b^	Reversal^c^	8	4	45	377	1	1	2	17	125	264	4,768	4,643	2	2	0	4
9049	Reversal	10	3	64	392	3	1	2	19	83	223	1,859	1,776	4	7	3	8
9051^b^	Reversal	8	4	61	387	3	2	4	20	218	251	4,724	4,506	5	5	1	9
9052^b^	Reversal	6	3	67	392	4	2	3	13	59	370	8,134	8,075	3	3	0	6
9002	PZP	19	6	34	381	0	0	0	11	2,206	*338*	8,635	6,429	0	7	0	7
9043	PZP	14	6	54	384	0	0	3	21	18	*338*	3,379	3,361	3	4	1	6
9055^b^	PZP	10	6	70	389	2	0	0	16	268	*338*	4,422	4,154	2	10	1	11
Red38^b^	PZP	19	6	23	392	0	0	1	8	540	*338*	3,808	3,268	1	4	0	5
9046^b,d^	Reversal	7	3	23	188	0	0	0	7	251	*338*	3,227	2,976	0	5	0	5
9010^b^	Reversal^c^	14	4	50	387	4	2	2	12	332	*338*	4,996	4,664	3	7	1	9
9028^b^	Reversal	10	3	61	384	1	1	1	21	172	*338*	4,923	4,751	1	10	0	11
9050^b^	Reversal	8	3	70	395	0	0	0	17	364	*338*	4,461	4,097	0	11	0	11
PZP-treated (*N* = 45)																	
$\bar{X}$		7.5	4.9	67.3	388.2	1.7	0.5	2.9	18.9	214.3	348.8	3,615.5	3,401.1	2.5	4.9	1.3	6.2
*SD*		4.5	1.1	14.1	11.9	1.9	0.8	3.4	5.1	322.3	101.3	1,678.2	1,565.2	1.8	1.9	1.4	2.0
Reversal (*N* = 15)																	
$\bar{X}$		9.1	3.3	61.2	369.9	2.1	0.9	2.9	17.1	195.3	297.8	4,178.2	3,982.7	2.9	5.9	2.3	7.3
*SD*		2.0	0.5	14.1	56.6	1.7	0.7	2.2	4.7	94.5	88.8	1,572.5	1,579.1	1.9	2.5	1.7	2.2

^a^Mean and *SD* values do not include 8 cows (bottom rows; below double line) for which individual baseline values could not be calculated. Therefore, *N* = 41 and 11, respectively, for porcine zona pellucida (PZP)-treated and Reversal cows for these variables.

^b^Cows with cattle mtDNA, based on Derr et al. (2012).

^c^No longer received PZP boosters after 2013. All other Reversal cows were last administered PZP boosters in 2012.

^d^#9046 died in December 2014 from a leg injury.
