# Supplementary material for: Effects of prolonged immunocontraception on the breeding behavior of American bison
Source: J Mammal. 2017 Aug 10;98(5):1272–87. doi: 10.1093/jmammal/gyx087 (PMC5901074; doi:10.1093/jmammal/gyx087)
Supplement: Supplementary Data SD1 [file gyx087_suppl_supplementary_data1.docx]

**Supplementary Data SD1.** — (A) Number of days of field sampling (total 145) and (B) number of bison observations or FP samples collected each month on Catalina Island from 1 June 2014 to 30 June 2015.


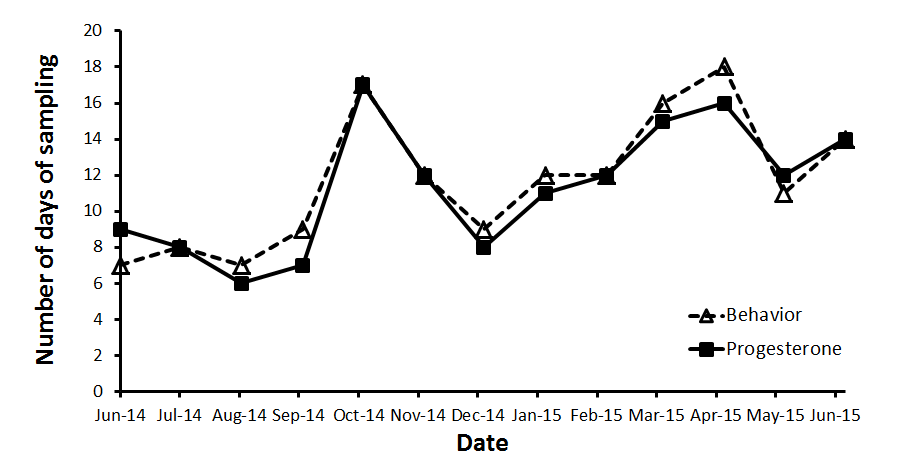

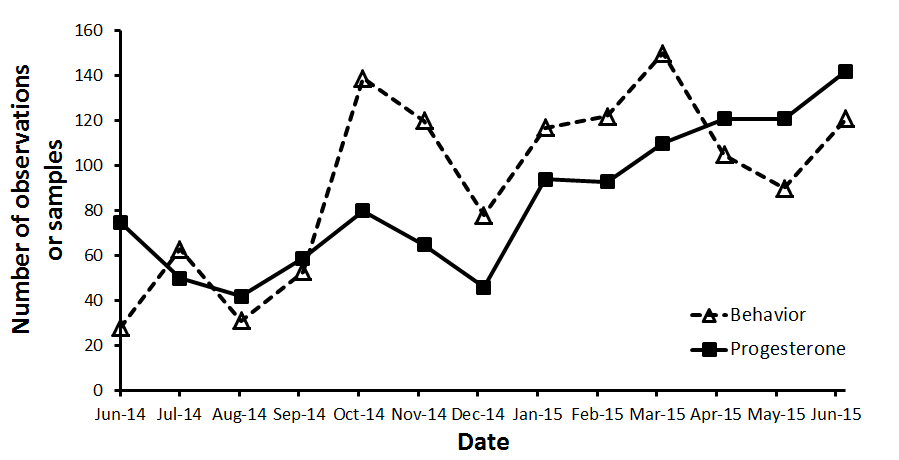


B

A
