# Supplementary material for: Effects of prolonged immunocontraception on the breeding behavior of American bison
Source: J Mammal. 2017 Aug 10;98(5):1272–87. doi: 10.1093/jmammal/gyx087 (PMC5901074; doi:10.1093/jmammal/gyx087)

**Supplementary Data SD2.**— Range of fecal progesterone values (FP, ng/g) for 60 bison cows on Catalina Island between 1 June 2014 and 30 June 2015. Open bars (green) are cows treated with PZP each year. Hatched bars (blue) are cows treated with PZP until 2012 or 2013 (Reversal cows). The line in the lower portion of each bar indicates the baseline FP value calculated following Brown et al. (2001). Eight cows (four PZP, four Reversal) for which a baseline could not be calculated are shown to the far right, with the dashed line (red) indicating the mean baseline FP value (338 ng/g) of the other 52 cows.


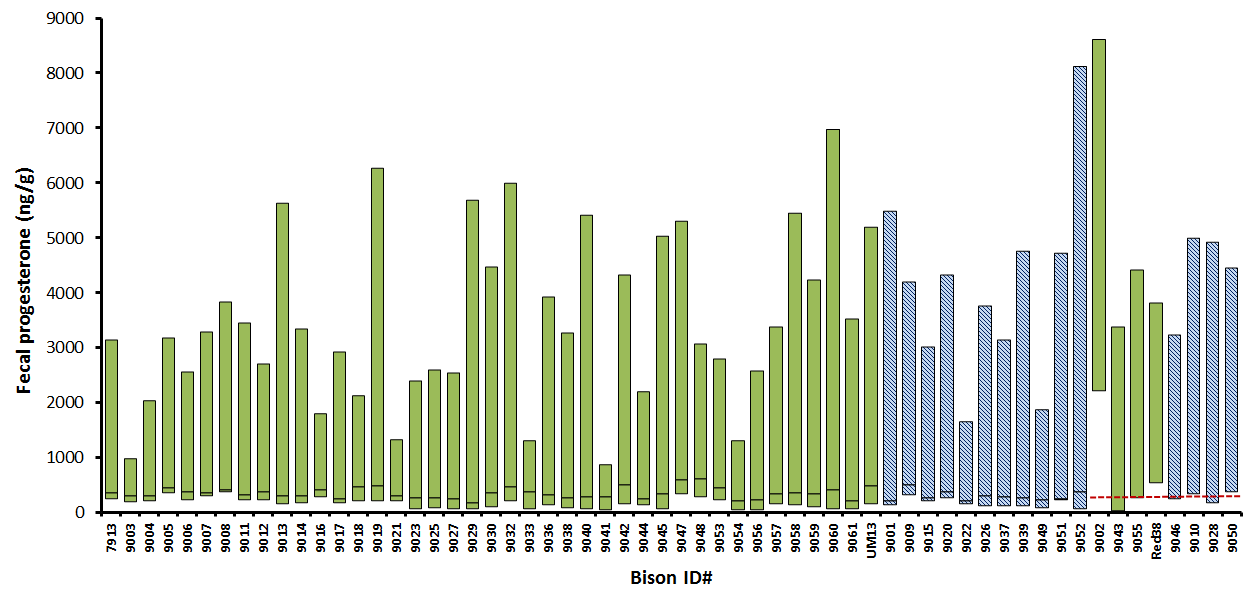

Supplement: Supplementary Data SD2 [file gyx087_suppl_supplementary_data2.docx]
